# It's the Economy! Perceptions of Host-Countries' Institutions and Individual Life Satisfaction of Intra-European Migrants

**DOI:** 10.3389/fsoc.2019.00042

**Published:** 2019-05-15

**Authors:** Irena Kogan, Jing Shen

**Affiliations:** ^1^School of Social Sciences, University of Mannheim, Mannheim, Germany; ^2^Mannheim Centre for European Social Research (MZES), University of Mannheim, Mannheim, Germany

**Keywords:** immigrant integration, life satisfaction, social comparisons, Europe, comparative

## Abstract

By drawing data from the European Social Survey (ESS) (2008–2016), this study explores how immigrants' assessments of economy, democracy, and quality of public goods (such as health and education systems) in the receiving societies contribute to their life satisfaction. Results indicate that satisfaction with the economy is the strongest correlate of individual life satisfaction among European immigrants, and this association is particularly pronounced among immigrants from Turkey, Eastern and Southern Europe. Assuming that immigrants compare institutions of their host and home countries when assessing institutional features of the host countries, relative gains in satisfaction with the performance of host-country economy are shown to be associated with particularly higher levels of overall life satisfaction among immigrants from Turkey, Eastern and Southern European countries than the rest of Europe. We conclude that, in relative terms, migrants from countries with less well-functioning economies to countries with more favorable economic conditions display higher levels of perceived satisfaction with the host country economies, which contributes considerably to their overall life satisfaction.

## Introduction

Whereas a vast body of migration research has examined objective indicators of immigrants' integration (see Van Tubergen et al., 2004 for comparative research; Kogan, 2006, 2007; Heath and Cheung, 2007; Heath et al., 2008 for a review; Fleischmann and Dronkers, 2010; Gorodzeisky and Semyonov, 2017; Khoudja and Platt, 2018 for recent research), immigrants' subjective evaluation of their life situation is far less studied. Recent years have, however, witnessed a growing interest in immigrants' own assessments of their lives in host societies, captured by individual life satisfaction (see Hendriks, 2015 for a review; Baykara-Krumme and Platt, 2018; Hendriks and Bartram, 2018; Kogan et al., 2018 for recent research). Conceptually, life satisfaction is defined as a result of evaluation, in the course of which individuals compare their perceptions of the actual situations with their hopes and expectations of how the situation should be, i.e., an ideal situation (e.g., Campbell et al., 1976; Shin and Johnson, 1978; Michalos, 1985 for a comprehensive overview). While factual situations can be largely approximated through individuals' economic, socio-demographic and socio-cultural circumstances, factors relevant to the description of the ideal situation seem to be more multifaceted and ambiguously defined. Among other things, an ideal situation depends on perceived norms, aspirations, past experiences, or on how immigrants compare themselves with others, such as relatives and friends in the country of origin or native-born colleagues and neighbors in the receiving country (Siegert, 2013).

In the current study, we explore how immigrants' assessments of the state of economy, democracy, and quality of public goods (such as health and education) in the receiving societies contribute to their life satisfaction. Unlike earlier research, which either focused exclusively on individual characteristics (e.g., Bartram, 2011) or predominantly on contextual determinants of the associations between immigrants' individual characteristics and life satisfaction (e.g., Hendriks and Bartram, 2016; Kogan et al., 2018), we examine how individual *perceptions* of host country structural conditions and institutional characteristics contribute to immigrants' overall life satisfaction. While addressing this research question, we pay particular attention to the differences in assessment of host country institutions across immigrant groups. A peculiarity of the current study is that we do not consider an immigrant's perception of the host-country context alone, but rather, we relate this perception to that of the immigrant's sending country^1^. To this end, we first compare an immigrant's perception of the receiving society with reports from similar individuals in the immigrant's country of origin. The underlying assumption is that had immigrants remained in their countries of origin they would have similar opinions as stayers with comparable characteristics in the respective countries. Even after moving to another country, immigrants would still maintain contacts in their countries of origin (via relatives or friends), and are likely to be influenced by opinions of their former fellow citizens. Alternatively, immigrants might also undertake comparisons within a reference frame in the countries of their current residence, i.e., with the native-born. In order to make immigrants and stayers/natives as much comparable as possible, in our empirical analyses, we match immigrants to stayers and the native-born, respectively, on a number of observed individual characteristics, such as age, gender, family status and educational level. Then we establish whether immigrants' assessments of host-country institutions diverge once these are compared to stayers and natives. Finally, we explore the association between assessments of host-country institutions, both absolute and relative, and individual life satisfaction with the aim to establish to what extent the choice of a comparison frame might be of relevance.

Before addressing the possible role of host country institutions and structural conditions and formulating related hypotheses, we describe patterns of migration within Europe, paying particular attention to driving forces behind migration flows and their implications for immigrants' subjective well-being. Subsequently, the data and methodological aspects of our study are presented, followed by the empirical analyses. We draw on the European Social Survey (ESS) data, a standardized comparative dataset on a large number of European countries, which meets key data requirement for our study: It contains identical information on movers and stayers with regard to core independent and control variables. The study concludes with the discussion of whether or not and how subjective perceptions of host-country performance shape individual life satisfaction, and what role potential comparisons with the sending countries might play in this regard.

### Patterns of Intra-European Migration

In order to better comprehend patterns of subjective well-being among European migrants, it is important to relate them to the history of intra-European migration, which since the Second World War has been marked by a number of major developments. These include guest worker migration and family reunification in the 1950–1970s, refugee migration to the West—predominantly from Eastern Europe (particularly up until the late 1980s), Yugoslavia (in the early 1990s) and Turkey—and finally migration within the European Union (as a result of freedom of mobility within the EU).

The phase of guest worker recruitment started in the mid-1950s, when North-Western European governments signed a number of bilateral agreements with peripheral and neighboring European countries. The main destination countries were Belgium, France, Germany, Luxembourg, the Netherlands, Sweden and Switzerland, whereas the main sending countries were Greece, Italy, Portugal, Spain, Turkey and Yugoslavia (Fassmann and Münz, 1992). In accordance with the Gravity model of migration (Zipf, 1946), in many cases geographical proximity of sending and destination countries played a major role in migration flows. For instance, Finland became a key source of labor force in Sweden; Irish went to the UK and Italians to Switzerland or Germany (Van Mol and de Valk, 2016). In accordance with macro-economic theories of migration (Harris and Todaro, 1970), migration was more likely to take place from economically less developed (regions of the) sending countries to more developed industrialized (regions of the) receiving countries and was primarily driven by economic considerations. Migrants from agricultural regions of Northern Portugal, Western Spain, Southern Italy, Northern Greece and Anatolia (Turkey) were pushed by scarce employment opportunities, low incomes and poverty (Bade, 2004) and pulled by abundant job opportunities in the lower segments of the labor market and higher living standards in Western and Northern Europe (see also Push-Pull-Paradigm by Lee, 1966 and dual labor market theory by Piore, 1979). Working abroad allowed migrants to accumulate financial resources and send remittances to their (extended) families, thereby contributing to higher consumption levels of those who remained in the sending countries. The oil crisis of 1973–1974 brought about halt in recruitment of guest workers and transformation of migration flows (Van Mol and de Valk, 2016). Instead of the circular patterns of labor migration, European countries started facing chain migration of the family members of migrants who had arrived in the framework of guest worker schemes (Fassmann and Münz, 1992; Hansen, 2002).

Apart from the former Yugoslavia, Eastern European countries were not a part of the 1950–1970s labor recruitment. Instead their migration to the West has been characterized by inflows of Eastern European refugees following political crises in Hungary (1956–1957), Czechoslovakia (1968–1969), and Poland (1980–1981) (Fassmann and Münz, 1992, 1994). The disintegration of the Soviet Union and the Yugoslavian wars in the early 1990s triggered a surge in numbers of asylum seekers and refugees within Western Europe and resettlement in Eastern Europe (Hatton, 2004; Van Mol and de Valk, 2016). In many cases, immigrants leaving Eastern European countries were not necessarily political refugees but ethnic minorities of the sending countries, who were able to relocate to their countries of ancestry after the fall of the Iron Curtain and the liberalization of travel (e.g., ethnic Germans predominantly from Poland, Hungary and the former Soviet Union; Ingrian Finns from Russia; a Greek minority from the former Soviet Union; and a Turkish minority from Bulgaria). The population groups were pushed by deteriorating political and economic situations in the sending countries and pulled by the prosperity and preferential treatment of the returning Diaspora members in the receiving countries.

The 1992 Maastricht Treaty's abolition of borders considerably eased intra-EU migration. It allowed European citizens to move freely within the EU and reduced many institutional barriers. Since the mid-1990s and particularly since the enlargement of the European Union to the East up until the recent economic crisis, fueled by the rapidly improving economic conditions, Southern Europe, Finland and Ireland—formerly major sending countries themselves—became magnets for immigrants, particularly from Eastern Europe. The recent global economic crisis brought about a revival of emigration from Southern Europe, as countries hit hardest by the crisis—Greece, Ireland, Italy, Portugal and Spain—again became emigration countries (Castles et al., 2014).

Population movements from the South to the North and from the East to the West dominated migration flows within Europe since the 1950s. Migrants heading from Turkey, Eastern and Southern Europe to Western and Northern European countries were pushed by scant employment opportunities and political unrest (particularly in Eastern Europe) in their home countries and pulled by more favorable economic conditions and promises of a better economic future abroad. Different migration motives among Western and Northern Europeans on the one hand, and Turks, Eastern and Southern Europeans, on the other hand might find reflection in immigrants' assessments of their life situation in the countries of their new residence.

### State-of-the-art Research and Hypotheses

Allardt's (1976) seminal approach “Having, Loving and Being,” which defines the role of the three factors in individual subjective well-being, can be seen as a conceptual framework for the current study. Whereas, “Having” captures material resources and basic living conditions, such as income, housing and basic (public) goods, “Loving” refers to the individual needs for social relations and emotional support and “Being” to the overall recognition, participation and feeling of belonging. “Having” forms a basis for the satisfactory functioning of an individual. Referring to differences between richer and poorer countries in the strength of the association between individual income level and life satisfaction reported by Easterlin (1973), Veenhoven (1997), Argyle (1999), and Böhnke (2008) suggest that satisfaction with basic needs is a precondition for other life domains, such as social approval and belonging. Indeed, research has shown that levels of life satisfaction are negatively correlated with unemployment levels (Clark, 2003) and positively associated with GDP per capita (Inglehart and Klingemann, 2000; Fahey and Smyth, 2004) of countries. These studies show that an individual's perceptions of structural conditions and how well a country's institutions function reflect not only the country's objective conditions, but more importantly, they could also capture individual evaluations that might be related to the individual's own needs and resources. Hence, such subjective perceptions can vary across individuals within the same country.

Pertinent research examining the role of institutional factors on life satisfaction posits that not only GDP and economic growth matter for subjective well-being, but also welfare state expenditures and political freedoms correlate with individual life satisfaction (Haller and Hadler, 2006). According to Diener and Suh (1999), citizens are more satisfied when they live in wealthy countries, which are characterized by high-quality education, health and legal systems. Addressing the role of host country institutions on immigrants' life satisfaction, Kogan et al. (2018) find that when taking into account the extent to which a host country is able to provide public goods, a country's wealth level does not seem to be particularly important for immigrants' life satisfaction, whereas a country's level of human development is associated with a higher life satisfaction of immigrants. Research on how specific aspects of the provision of public good shape individual life satisfaction is scant (for some exceptions see Hsieh, 2017, on the relationship between quality of homecare service and quality of life).

As for the “Being,” Dorn et al. (2008) contend that citizens' well-being may be enhanced by their participation in the political decision-making processes and by the perceived extent of the procedural fairness during the processes. It is plausible that citizens' empowerment through democratic institutions should lead to higher levels of self-reported life satisfaction. Whereas Frey and Stutzer (2003) report that direct democracy (as in Switzerland) is significantly associated with levels of happiness in this country, evidence from other international cross-sectional research does not unanimously confirm such a relationship (Schyns, 1998; Inglehart and Klingemann, 2000; Veenhoven, 2000; see also Dorn et al., 2007, 2008).

Altogether, existing research has underscored the importance of countries' economic and political conditions as well as quality of public goods for individual well-being. It has also been shown that, alongside examining the role of political and institutional settings, researchers should pay attention to the individual perceptions of institutions that correlate with life satisfaction beyond objective measures of the quality of society (Böhnke, 2008). To date, no study has specified which institutions would be perceived as most important for immigrants' well-being and hence contribute the most to immigrants' assessments of their life satisfaction. We hypothesize that due to the predominantly economic nature of the intra-European migration and the improvement of economic well-being as the foremost migration motive, immigrants' satisfaction with the state of economy should be the strongest correlate of their overall life satisfaction (*Hypothesis 1*).

Given that the association between perceptions of country's (economic) performance and individuals' overall life-satisfaction exists, the question arises, whether it is uniform for groups of inter-European migrants. Attribution mechanism, prominent in psychology (Jones and Davis, 1965; Kelley, 1971) would suggest that people growing up in more developed countries should have a weaker tendency than their counterparts growing up in less developed countries to associate their life satisfaction with the external environment. Research indeed shows that individuals originating in societies with established democratic institutions and well-functioning economies seem to be more likely to decouple their assessments of personal life situation from the satisfaction with national affairs (Andrews and Withey, 1976; Böhnke, 2008). In contrast, personal satisfaction might be much more strongly attached to one's view of the country among individuals facing lower living standards and limited political freedoms for a large part of their lives. Therefore, when moving away from their homelands, immigrants from such countries should put more emphasis on the host-country conditions when evaluating gains or losses of migration. This allows us to postulate that satisfaction with the state of economy should play a particularly important part in the overall life satisfaction among immigrants originating from countries with less well functioning labor market institutions, i.e., those coming from Turkey, Eastern and Southern Europe (*Hypothesis 2*).

From the standpoint of the social comparison theory (Festinger, 1954), immigrants' attachment to both the places where they originate from and where they currently live, is likely to result in multiple frames for comparison. Socialized in the country of origin, immigrants tend to compare their current situation to the situation in the sending country. Such comparisons might be further nurtured by social contacts in the country of origin, i.e., through conversations with their friends and relatives who remained in the sending country. Finally, media might keep immigrants' comparisons with sending countries alive even if migration lays back in time. If someone comes from a country with institutions functioning less efficiently than in the host country, perceptions of host country institutions might be positively biased and hence potentially be more positively associated with the overall life satisfaction. An opposite case is, in principle also possible: when someone originates from a country with healthy functioning institutions but emigrates to a country, where social structures function less well, his/her perceptions of host-country institutions would be downward biased and potentially reflected in the overall life satisfaction. Individuals migrating from Turkey, Eastern and Southern Europe should on average experience greater improvement in their economic lives than those moving within the rest of Europe. Based on this, we hypothesize that the association between the relative level of satisfaction with host country institutions, and particularly economy, and the overall life satisfaction among these immigrants should be particularly high compared to the respective association among immigrants originating in the rest of Europe *(Hypothesis 3*).

### Data and Measurements

The analyses are based on the ESS cumulative data^2^ for the years 2008–2016 (waves 4–8), thus covering the period during and in the aftermath of the recent economic crisis. The ESS enables a truly comparative European perspective, as strong efforts have been made to ensure comparability across the participating countries. We concentrate on the European migration in and from the following 30 countries: Austria, Belgium, Bulgaria, Switzerland, Cyprus, Czech Republic, Germany, Denmark, Estonia, Spain, Finland, France, UK, Greece, Croatia, Hungary, Ireland, Iceland, Italy, Lithuania, Luxembourg, Netherlands, Norway, Poland, Portugal, Russia, Sweden, Slovenia, Slovakia, Turkey and Ukraine^3^. The focus on solely European migrants within European countries is justified by the design of the study, which requires identical information on both migrants and stayers not only regarding their individual characteristics (such as socio-demographics) but also with respect to their assessments of countries' institutions. Immigrants are defined in our study as individuals who were born in countries other than their current country of residence and arrived to the country of their current residence in years 1955–2017. Individuals who reside in their birth countries throughout their lives are referred to as stayers. In additional analyses, we also refer to patterns of life satisfaction among the native-born in host countries, who are defined as residents, born on the territory of the respective country. We further compare satisfaction with the performance of host-countries among immigrants and the population born in these countries.

Our dependent variable is the level of life satisfaction, which is asked in all waves of the ESS: “All things considered, how satisfied are you with your life as a whole nowadays?” The answer categories range from 0 (extremely dissatisfied) to 10 (extremely satisfied), resulting in an 11-point Likert-scale. A consistent measurement and standardized formulation ensures comparability across the ESS waves and the countries participating in the survey. Although we acknowledge that the multifaceted character of subjective well-being is possibly captured better by multiple indicators, a detailed information on different aspects of subjective well-being is not available in the ESS.

Since we focus in particular on the comparison of immigrants' current situation in the receiving country with a hypothetical situation in the sending country if they had remained there or had been influenced by their relatives and friends residing in their country of origin, we match immigrants with stayers on the basis of their core socio-demographic characteristics. The procedure is the following. In the first step, based on the OLS regression analyses, we predict the value of satisfaction with economy, democracy, the state of the education and health systems for a stayer with each possible combination of the following variables: gender, age (with the following age groups: 15–24, 25–39, 40–59, and 60–65), marital status (married vs. other), presence of children (yes vs. no), educational level (lower secondary or below, upper secondary, or post-secondary and tertiary), country and ESS round. In the next step, each immigrant is matched with a stayer based on the set of above-mentioned characteristics.

Since the major focus of the study is on the association between immigrants' satisfaction with the functioning of host country institutions (in both absolute and relative terms) and their general life satisfaction, our central independent variables pertain to individuals' satisfaction with the functioning of the country in which they reside, including satisfaction with the provision of public goods (health services and education systems), the economic situation and the state of democracy. Each of these variables is measured on an 11-point scale ranging from 0 (extremely dissatisfied) to 10 (extremely satisfied). In addition to the absolute levels of life satisfaction with host country institutions, we also focus on the relative levels. These are calculated as a difference between the level of life satisfaction with respective host country institutions and the level of life satisfaction with institutions of the sending country among socio-demographically identical stayers (based on the above-mentioned set of characteristics). The theoretical range of the newly created relative levels of life satisfaction with host country institutions is between −11 and +11, whereby positive values pertain to higher satisfaction with host country institutions than with sending country institutions and 0 pertains to equal level of satisfaction with institutions of both countries. To test whether individual assessments of host country institutions are differently associated with overall levels of life satisfaction depending on the source country of immigrants, we include interaction terms between domain-specific levels of life satisfaction and immigrant origin groups (see below for the definition of these groups).

At the individual level, a set of demographic, socioeconomic and migration-specific characteristics is included. Demographic traits, such as age and its quadratic term, gender, number of persons in the household and (ever) having children, are controlled. We measure socioeconomic characteristics with the following variables: employment status—coded as a categorical variable with three groups: employed, unemployed and inactive (with “inactive” used as a reference category)—, years of schooling, and the relative position of the household income in the income distribution of the corresponding host country (measured in deciles). We include individuals with missing information on income by assigning them to the modal income decile of the host country's corresponding income distribution. We use a dummy variable to distinguish cases with missing income information.

Migration-specific characteristics mainly refer to immigrants' countries of origin, the length of residence in the host country after immigration, language use and citizenship acquisition. On the basis of the countries of origin, we differentiate between immigrants from (1) Eastern Europe, (2) Northern Europe, (3) the UK and Ireland, (4) continental Europe, (5) Southern Europe and (6) Turkey. The country or region of origin not only indicates an immigrant's cultural background, but also serves as the reference for immigrant evaluations of their post-migration situation. An obvious question is whether the classification of origin groups is meaningful and valid. Our sensitivity analyses, in which we reran all analyses while excluding one country from each origin group at a time yielded comparable results for all origin groups, but one. The analyses for the UK/Irish groups seem to be driven by the UK data. Once excluding the UK, the coefficients for the perception of economy and democracy increase substantially. Apparently, for Irish immigrants perceptions of economy and democracy are stronger associated with life satisfaction than any other immigrant group in the dataset. Having Irish immigrants as a separate group is, however, unwarranted due to a relatively low sample size of the group. Hence, we decided to stick to the group of English-speaking immigrants, but refer reader to the differences between Irish and British immigrants when applicable.

Length of residence in the host country is captured by the variable “years since migration” (YSM) and coded as a categorical variable with four groups (residing in the host country for 0–5, 6–10, 11–20, and above 20 years; here, the group residing in the host country for 5 years or less is used as a reference category). Speaking the host country's national language at home—the only indicator of language proficiency available in the ESS data—is used as a proxy for the degree of cultural assimilation. Citizenship status of the host country is another indicator of integration—this time legal integration—in the host society.

In addition, we control for variables that are commonly mentioned in the existing literature on life satisfaction (see Kamberi et al., 2015 for a summary). For example, religiosity has often been considered a factor potentially protecting individuals in difficult life situations. We measure religiosity on an 11-point scale ranging from not at all religious to very religious. Immigrants' minority status is captured by a dummy variable, indicating whether immigrants mention to belong to an ethnic minority group. Individual health status is measured by immigrants' subjective assessment of their health situation, ranging from (1) very bad to (5) very good. We further include a variable “feeling of safety when walking alone in the local area after dark,” which indicates whether one feels safe in the neighborhood. The variable's range is from (1) very unsafe to (4) very safe. We take into account the degree of immigrants' sociability by including the variable measuring how often they meet with friends, relatives and colleagues, with answer categories reaching from (1) never to (7) every day. In addition, we control for the survey waves to capture a general time trend in life satisfaction. Finally, we include host country fixed effects, so that our results pertain to the difference in life satisfaction among immigrants residing in the same receiving country. In doing so, we control for differences in country-specific levels of life satisfaction as well as for institutional influences able to shape individual life satisfaction. Distributions of the independent and control variables are available upon request.

The importance of the perceptions of various host country characteristics and their patterns of association with overall life satisfaction might obviously differ depending on the population analyzed and the objective conditions. To homogenize the analyzed population, we restricted our analyses to working-age immigrants (aged 15–65).

### Descriptive Results

**Figure 1:**
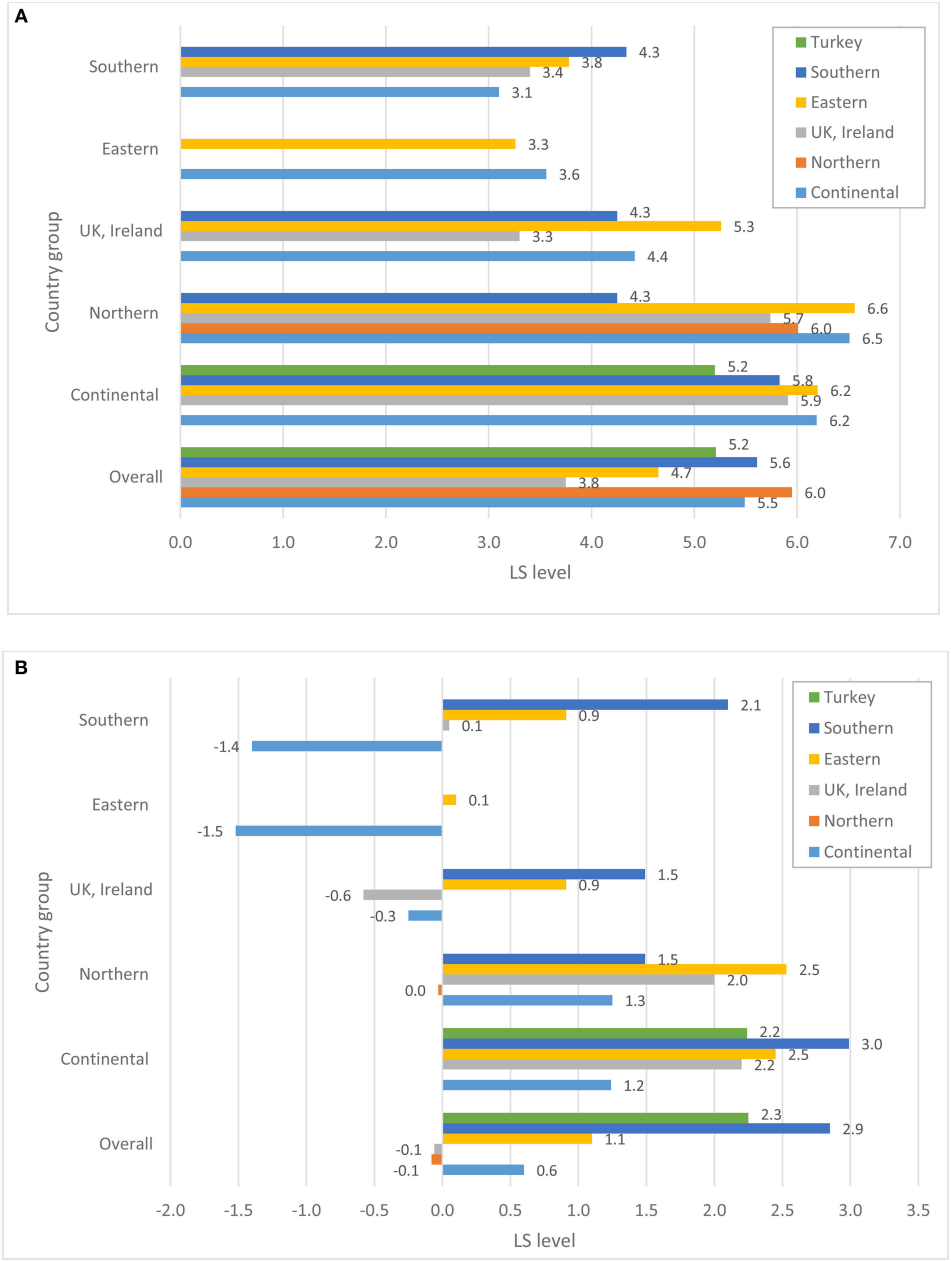
**(A)** Satisfaction with the state of economy, absolute level. **(B)** Satisfaction with the state of economy, relative to stayers in the country of origin. Source: ESS 2008-2016 (rounds 4-8), weighted data, authors' calculations.

**Figure 2:**
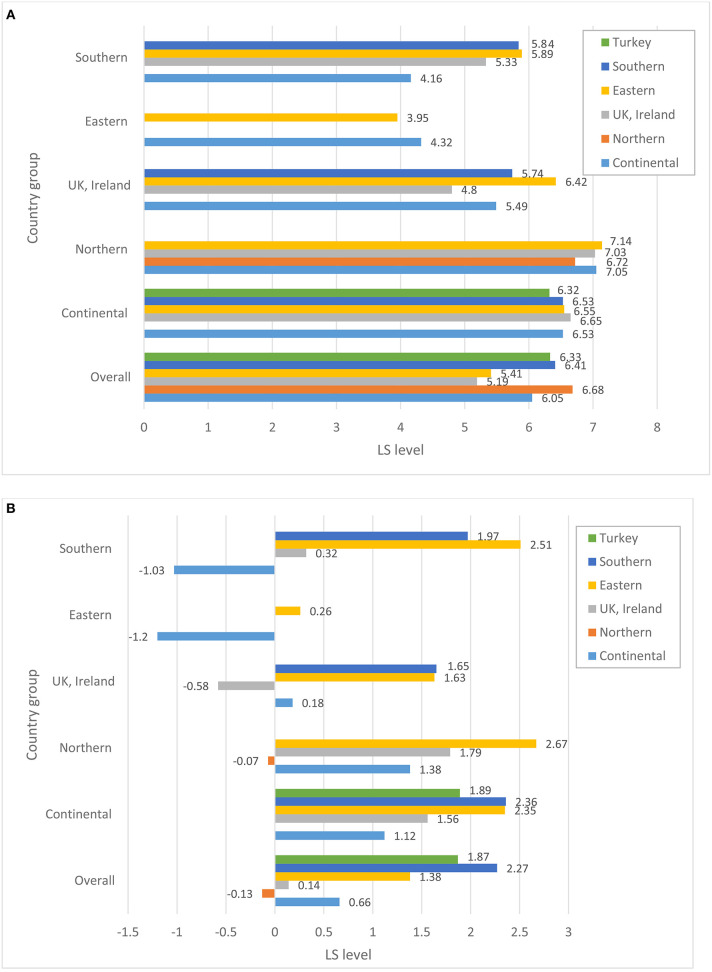
**(A)** Satisfaction with the state of democracy, absolute level. **(B)** Satisfaction with the state of democracy, relative to stayers in the country of origin. Source: ESS 2008-2016 (rounds 4–8), weighted data, authors' calculations.

**Figure 3:**
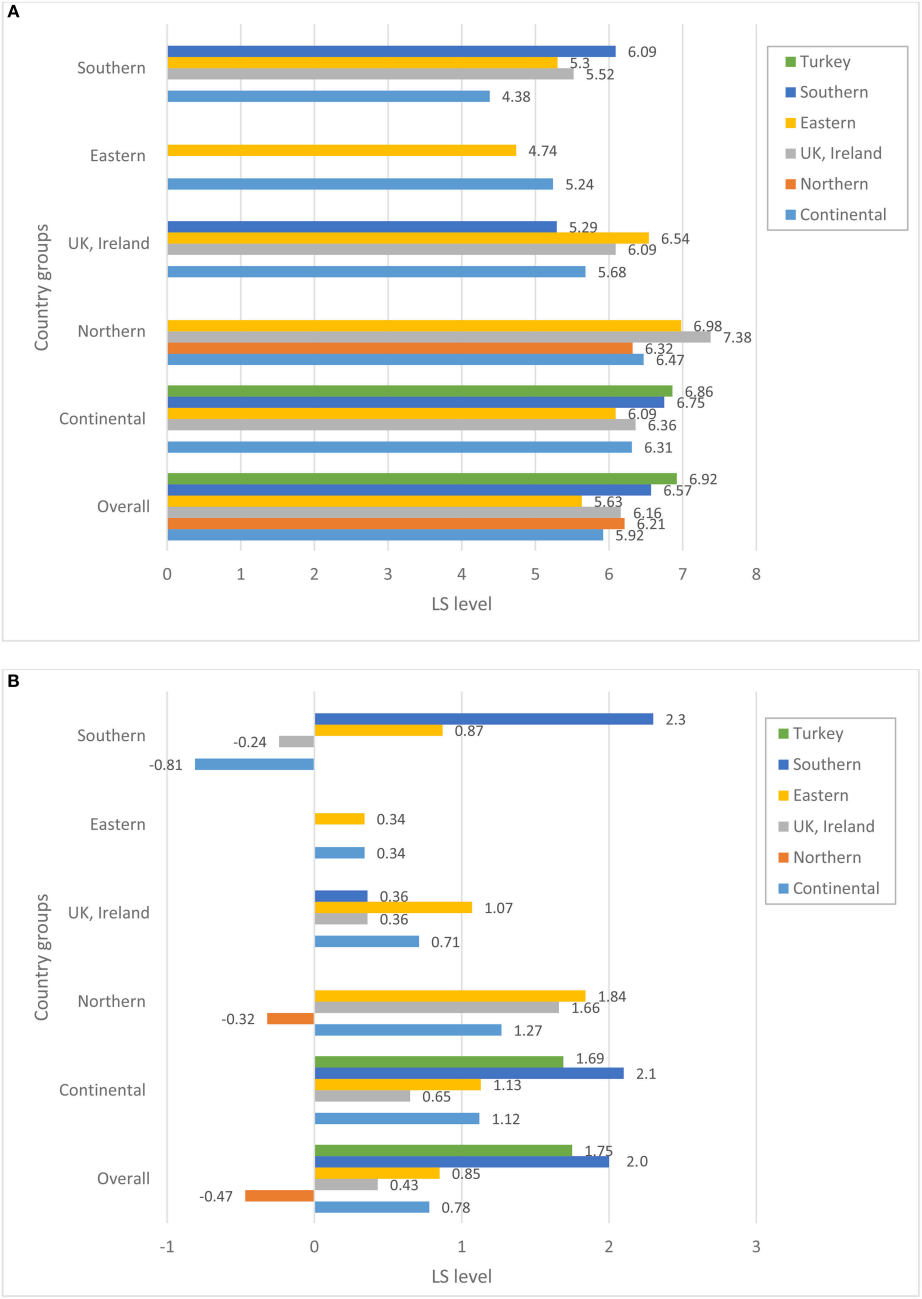
**(A)** Satisfaction with the state of education system, absolute level. **(B)** Satisfaction with the state of education system, relative to stayers in the country of origin. Source: ESS 2008-2016 (rounds 4–8), weighted data, authors' calculations.

**Figure 4:**
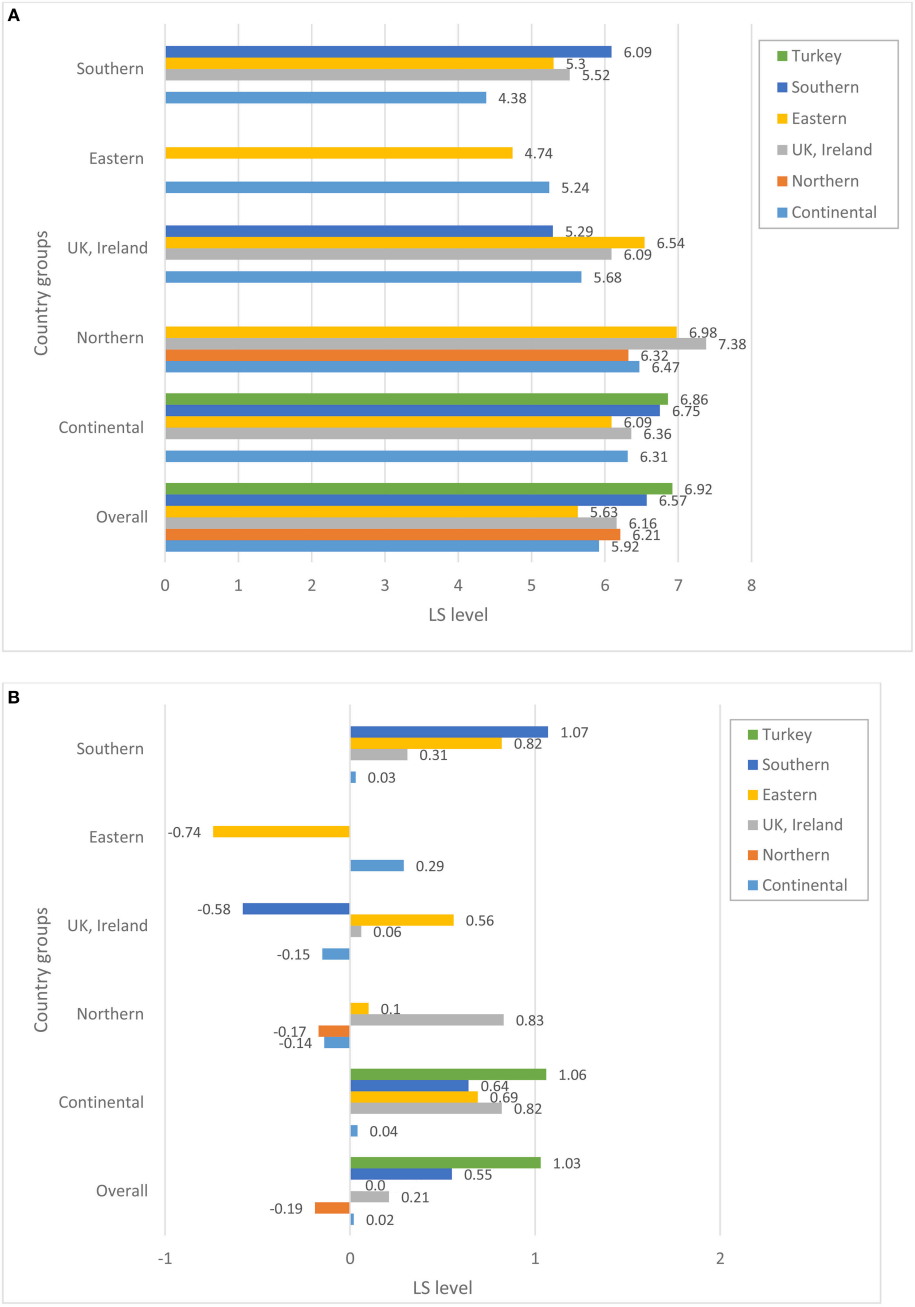
**(A)** Satisfaction with the state of health system, absolute level. **(B)** Satisfaction with the state of health system, relative to stayers in the country of origin. Source: ESS 2008-2016 (rounds 4–8), weighted data, authors' calculations.

Before turning to the patterns of association between perceptions of host-country economic conditions, state of democracy, and public services and the individuals' overall life satisfaction, we explore whether immigrants from various source regions differ in terms of their satisfaction with host country structural and institutional conditions. Figures 1–4 plot group average levels of satisfaction with the state of economy (Figure 1), democracy (Figure 2), education system (Figure 3), and health services (Figure 4) in both absolute (panel a) and relative (panel b) terms. The figures show average levels of satisfaction with each of four contextual characteristics for an immigrant regardless the country s/he resides in (labeled “overall”) and separately by groups of destination countries. Similar to groups of origin, we differentiate between continental, Northern, Eastern and Southern European countries, as well as UK and Ireland.

Regarding satisfaction with the state of the host country economy in general, considerable variation is observed across the origin groups. Overall, Northern Europeans—compared to other immigrants—seem to be the group that is most satisfied with the state of economy and democracy and highly satisfied with the education system and health services in their countries of residence. Immigrants stemming from the UK and Ireland are, on the other hand, the least satisfied with the state of economy and democracy in their host countries. East Europeans are one of the groups with consistently low levels of satisfaction in all domains of host country institutions compared to the rest. Southern Europeans, on the other hand, are among the most satisfied immigrants regarding all institutional characteristics of the host countries they reside in. A similar observation can be made for immigrants originating in Turkey.

A closer look at the combinations of origin and destination countries helps to better understand where the average patterns come from^4^. Not surprisingly, satisfaction with host-country institutions largely depends on the country an immigrant resides in. Practically all immigrant groups are more satisfied with the state of economy when they live in Northern and continental countries, and least satisfied with institutions in Eastern Europe. Still, we observe some variation across ethnic groups residing in the same region: whereas immigrants from continental and Eastern European countries are rather positive about the economy of continental countries, Turkish immigrants are substantially less satisfied. On the other hand, Turkish immigrants appear to be mostly satisfied with the state of education and health system in continental Europe and are not different from the rest when assessing the state of democracy. This indicates that assessments of host-country institutions do not just reflect objective conditions in the pertinent country, but are carried out through the lenses of individuals' socio-economic status, needs and experiences. The disparities in the assessment can be substantially large, as indicated, for example, by the levels of satisfaction with economy among Eastern and Southern Europeans in Northern Europe regarding economy, or Southern Europeans and immigrants from continental Europe regarding the health system. Overall, opinions diverge more when it comes to the assessment of the host-country economy than any other institutions. Immigrants are particularly unanimous regarding lower levels of assessments for Eastern European destination countries.

But how immigrants evaluate host-country institutions if they were to compare them with the institutions back in their countries of origin? Relative to the situation in their source countries, the groups that are the most satisfied with the host country institutions are Turks, Southern and—to somewhat lesser degree—Eastern Europeans. Referring to the bars capturing the “overall” level of relative satisfaction (see Figures 1B–4B), all three groups are consistently more satisfied than the rest with economy, democracy and education system, and partially also with health services when comparing them to the institutions of their home countries^5^. Immigrants from Nordic countries, Ireland and the UK, on the other hand, assess the state of economy and democracy in their source and host countries rather similarly, under the assumption that their opinions about source countries would coincide with those of the socio-demographically similar stayers. Regarding the health and education systems, their opinions diverge: whereas immigrants from Nordic countries evaluate systems of public goods in the host country less favorably, Irish and British favor education and health systems in their host countries more than in their source countries. Immigrants from continental countries, on average, are more satisfied with institutions of the host country compared to those of their source countries; a single exception is health system. Taken together, this suggests that Turkish, Southern and Eastern European immigrants and, to a somewhat lesser degree, immigrants from continental Europe improve their utility compared to stayers back home, whereas Northern Europeans do not, at least not regarding institutional domains of the host-country featured in this study.

A look at the combination of countries of origin and destination reveals a more differentiated picture. Immigrants from the UK and Ireland, who are found in sufficiently large numbers in all destination countries apart from Eastern Europe, seem to be more satisfied with the state of economy and democracy if they reside in continental or Northern European countries, but not otherwise. Immigrants from continental Europe are satisfied with the state of economy, democracy and public services if they reside in other continental or Nordic country, but are substantially dissatisfied with economy and democracy (relative to their source country) in Eastern and Southern European countries. Gains in satisfaction with economy are the largest for Southern Europeans when they reside in continental Europe, but are much lower once they live in the UK, Ireland or Northern Europe. Eastern Europeans are more satisfied with institutions of any destination country than their home country, with one exception when they reside in another Eastern European country. Their satisfaction with the health system in another Eastern European country seems to be particularly low.

**Figure 5:**
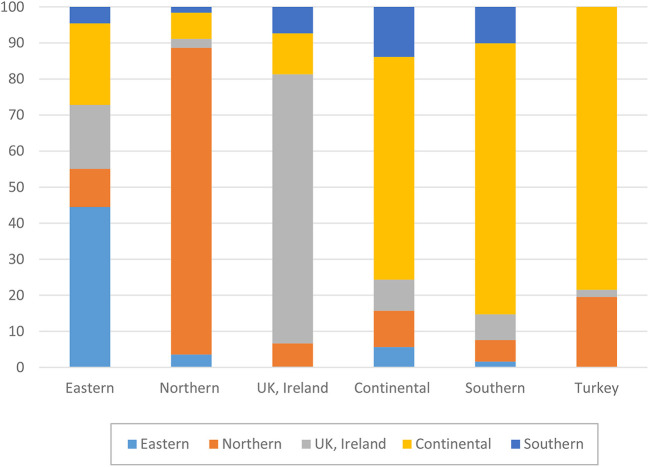
Composition of immigrants in European receiving regions by origin. Source: ESS 2008-2016 (rounds 4–8), weighted data, authors' calculations.

The fact that overall averages deviate from country-of-residence averages implies that we should definitely consider differences in the distribution of origin groups across destination countries. Thus, if we look at the total pool of immigrants (see Figure 5) we immediately notice that, for example, the bulk of immigrants from Nordic countries reside in other Nordic countries. Similarly, a large majority of Irish and British immigrants reside in Britain or Ireland, respectively. Obviously, if these immigrants were to compare the institutions of their host countries with those of their sending countries, they might not find any substantial differences. This might be different for Turks, Eastern and Southern Europeans. The bulk of Southern European migrants reside in the continental European countries, whereas only a small share of them live in other Southern European countries. Among Turkish immigrants, the two key destinations are continental and Northern Europe. It is fair to assume that Turks and Southern Europeans residing in wealthier countries of Europe might be particularly satisfied with the functioning of institutions in these countries once comparing them to those in their sending regions. The case of Eastern Europeans is somewhat different. Almost a half of all Eastern Europeans reside in other Eastern European countries, but a substantial proportion of them is found in the continental European countries, Ireland and the UK. Similarly, immigrants from continental countries reside in various destinations, with about a half of them settling in another continental country. So, it is not surprising that for these two groups, we find a large variation in assessments of host-country institutions, with Eastern Europeans being rather satisfied, for example, with economy in Nordic countries, but not in another Eastern European destination, or immigrants from the continent being satisfied in another continental country, but not in Ireland or the UK (cf. Figure 1B).

### Multivariate Results

Descriptive results presented in Figures 1A,B−4A,B demonstrate substantial variation in levels of satisfaction with institutions across immigrant groups, overall and separately by destination. We also acknowledge differential settlement patterns among immigrants in European countries in Figure 5. The multivariate analyses presented in this section will take the heterogeneity of immigrants' destinations by means of host country fixed effects. In such a way, our models seek to fully control for host country–specific factors, thus making it possible to compare the perceptions of institutions among immigrants residing within the same country. Table 1 reports selected coefficients from the OLS regression models predicting overall life satisfaction as a function of satisfaction with host country institutions (state of democracy, health services and education system) as well as satisfaction with economic situation, while controlling for an extensive set of individual background characteristics described in the methods section. Whereas the results in the four left columns pertain to the absolute measures of area-specific life satisfaction, the results in the last four columns report respective findings concerning relative measures of life satisfaction, i.e., satisfaction with host country relative to source country institutions (as they are reported by socio-demographically comparable stayers). In order to estimate the statistical significance of the coefficients of area-specific satisfaction for every origin group directly, we ran each model 6 times by using each of the respective origin groups as the baseline. Consequently, coefficients in Columns 1 and 5 directly indicate both the magnitude and statistical significance of the b-coefficient pertaining to the perceptions of host country institutions in life satisfaction for each respective origin group. Standard errors and beta coefficients are found in columns 2–3 and 6–7, respectively. Columns 4 and 8 document whether the coefficients pertaining to the group in question are statistically significant from another group, indicated in the table by a letter. For example, “the effect” of the absolute level of satisfaction with the state of economy on the overall life satisfaction is significantly different for immigrants from continental and Northern European countries. Regarding the relative levels of satisfaction with economy, the “effects” for continental European immigrants are different from those found among immigrants from Eastern and Southern Europe as well as Turkey. The sample size for the analyses of the relative satisfaction with institutions is somewhat lower due to some missing matches in the stayer sample. This might hinder the direct comparability of the coefficients across models with absolute and relative satisfaction with host country institutions. Restricting the sample size in the models of absolute measures of satisfaction to the cases used in the analyses of the relative measures of satisfaction yields rather similar patterns (results are not shown but available upon request).

**Table 1:** Selected coefficients from OLS regressions predicting life satisfaction among immigrants in Europe arriving since 1955.

	**Absolute**				**Relative to stayers in the country of origin**			
	**b**	**se**	**beta**	**Sign. dif**.	**b**	**se**	**beta**	**Sign. dif**.
**(A) CONTINENTAL**								
State of economy	0.23^***^	(0.04)	0.27	b	0.15^***^	(0.03)	0.19	d, *e*, f
State of democracy	0.05	(0.03)	0.05		0.03	(0.03)	0.03	c
State of education	0.01	(0.03)	0.01		0.00	(0.03)	0.00	
State of health system	0.08^**^	(0.03)	0.09		0.10^***^	(0.03)	0.13	*c*, e
**(B) NORTHERN**								
State of economy	0.12^*^	(0.05)	0.14	a, d, e, f	0.10^*^	(0.05)	0.13	d, e, f
State of democracy	0.07	(0.05)	0.08		0.10^*^	(0.05)	0.12	
State of education	0.04	(0.05)	0.05		0.02	(0.04)	0.02	
State of health system	0.05	(0.05)	0.05		0.05	(0.05)	0.06	
**(C) IRELAND/UK**								
State of economy	0.17^***^	(0.04)	0.20	d, *e*, f	0.15^***^	(0.04)	0.18	d, e, f
State of democracy	0.13^***^	(0.04)	0.15	*a*	0.13^***^	(0.04)	0.15	a*, d*,
State of education	0.08^*^	(0.04)	0.08		0.07^+^	(0.04)	0.08	
State of health system	0.01	(0.03)	0.02	*d*	0.03	(0.04)	0.04	*a*
**(D) EASTERN**								
State of economy	0.28^***^	(0.02)	0.33	b, c	0.24^***^	(0.02)	0.30	a, b, c
State of democracy	0.09^***^	(0.02)	0.10		0.05^**^	(0.02)	0.06	*c*
State of education	0.05^*^	(0.02)	0.05		0.05^*^	(0.02)	0.05	
State of health system	0.07^***^	(0.02)	0.09	*c*	0.07^***^	(0.02)	0.09	
**(E) SOUTHERN**								
State of economy	0.26^***^	(0.04)	0.31	b, *c*	0.24^***^	(0.04)	0.29	*a*, b, *c*
State of democracy	0.06^+^	(0.04)	0.07		0.05	(0.04)	0.06	
State of education	0.07^+^	(0.04)	0.07		0.04	(0.04)	0.05	
State of health system	0.03	(0.04)	0.04		0.00	(0.04)	0.00	a
**(F) TURKEY**								
State of economy	0.28^***^	(0.04)	0.33	b, c	0.29^***^	(0.04)	0.36	a, b, c
State of democracy	0.12^**^	(0.05)	0.13		0.09^*^	(0.04)	0.11	
State of education	0.05	(0.05)	0.05		0.01	(0.05)	0.01	
State of health system	0.07^+^	(0.04)	0.08		0.06	(0.04)	0.08	
*N*	5,292				5,100			
*R*^2^	0.39				0.37			

*Source: ESS 2008-2016 (rounds 4-8), weighted data, authors' calculations*.

*(1) ^+^p < 0.10*,

* *p < 0.05*,

** *p < 0.01*,

*** *p < 0.001; (2) Letters in column 4 and 8 indicate whether differences of the group shown are significant compared to the groups indicated by a letter; letters in bold pertain to coefficients significant solely at 10%-level. (3) Control variables included in the model are: age, age squared, gender, family status, presence of children, employment status, YSM, citizenship status, income, language spoken, social contacts, safety situation, subjective health, religiosity, minority status, origin groups main effects, country of residence fixed effects, year of interview fixed effects*.

A close look at the first column in Table 1 suggests that satisfaction with economy contributes significantly to the overall life satisfaction across all origin groups, and the magnitude of the respective coefficients is much higher than when it comes to the satisfaction with the state of democracy, education system, or health services. This clearly accords with our first hypothesis.

Alongside this general pattern, there are some host country group differences that require particular elaboration. Satisfaction with economy contributes least to the overall life satisfaction among immigrants from Northern Europe and the difference to other groups (apart from those coming from Ireland and the UK) is statistically significant. Among Eastern and Southern Europeans as well as Turks, satisfaction with the economy contributes substantially to the overall life satisfaction, and the difference to immigrants from Nordic countries, UK and Ireland is statistically significant. Among immigrants from continental countries the state of economy plays a considerable role in the overall life satisfaction, the coefficient is, however, statistically different only if compared to the one found among Northern European immigrants. All in all, this supports the second hypothesis, which expects perceptions of economy to play a particularly strong role in life satisfaction of immigrants from Turkey, Southern and Eastern Europe. For these immigrants, perceptions of economy contribute to the overall life satisfaction both substantively and statistically (with a single exception of the comparison to immigrants from continental countries) stronger than it is the case of other immigrants.

Perceptions of the state of democracy contribute significantly to the overall life satisfaction among immigrants from Ireland/UK, Turkey and Eastern Europe. These coefficients are, however, largely not statistically different across various immigrant groups^6^. Perceptions of the state of education system contribute significantly to the overall life satisfaction among immigrants in Ireland/UK and Eastern Europe, but here again, we observe no significant difference across immigrant groups in the strength of association between evaluations of country's education system and individual life satisfaction. Satisfaction with the state of health services significantly matters for the overall life satisfaction among immigrants from continental and Eastern Europe, but the respective coefficients are not statistically different from the ones pertaining to other immigrant groups.

But what if we assume that immigrants compare themselves with the stayers in their origin countries when evaluating host country institutions? Does this alternate the association between satisfaction with institutions in the host country and overall life satisfaction? The answer can be found in columns 4–8 of the same table. Results clearly indicate that immigrants from Turkey, Southern and Eastern Europe attach higher relative importance to a favorable economic situation in the host country (vs. home country) in their overall life satisfaction compared to immigrants originating in other European countries. In other words, relative satisfaction levels with the state of economy contribute substantially more to these immigrants' overall life satisfaction. Satisfaction with economy matters also for the life satisfaction of immigrants from continental and northern Europe, Ireland and the UK, but the respective coefficients are of much lower magnitude. Moreover, they tend to differ from the coefficients related to the absolute levels of satisfaction with the state of economy, particularly among immigrants from continental countries. Other patterns are much in line with the results pertaining to absolute levels of satisfaction with host country institutions presented in columns 1–4.

In sum, the findings for European immigrants residing in other European countries largely confirm our first hypothesis about European immigrants of working age attaching larger importance to satisfaction with economy once defining their overall levels of life satisfaction. Immigrants from Turkey, Southern and Eastern Europe display higher levels of association between both absolute and relative levels of satisfaction with economic performance of the host country and the overall life satisfaction. Both findings concord with the second and third hypotheses.

In a set of additional analyses (see Appendix Figures A.1–A.4), we ask a related question, how immigrants' perception of host-country structural conditions and institutions compare to those of the native-born and whether they are uniquely associated with individual life satisfaction. A variable, pertaining to the difference in the level of satisfaction with the state of economy, democracy and public goods between immigrants and comparable native-born in host countries is created similarly to the variable capturing difference between immigrants and stayers in immigrants' home countries. Our results indicate that immigrants tend to be overall similarly or more satisfied with the functioning of host-country economy, democracy and systems of public goods compared to the native-born population in these countries. Whether this is an indication of particular immigrant optimism or a manifestation of realized mobility aspirations needs to be explored in the future. Still there are exceptions to the general trend of positive assessment of host-country institutions. Thus, Eastern European immigrants residing in Eastern European countries other than countries of their birth appear to be less satisfied with the state of economy, democracy and health system compared to the natives. British and Irish immigrants are less satisfied with the economy in Nordic countries than the natives of these countries. UK/Irish and immigrants from Southern European countries are less satisfied with education system in Southern Europe, whereas immigrants from Nordic countries are less favorable about the education system once they reside in another Northern European country. The differences in life satisfaction between immigrants and comparable natives are, however, smaller in magnitude than the respective differences in hypothetical comparisons with the stayers.

Results of the multivariate analyses (Table A.2) deliver a picture, which largely resembles patterns of association between the absolute levels of satisfaction with host country institutions and individual life satisfaction. Still, the main message holds: economy matters most and it matters stronger for immigrant groups, which emigrated from areas marked by lower levels of economic development.

### Summary and Discussion

In recent years, the European continent has experienced a considerable rise in migration. Whereas, a large proportion of newcomers are immigrants and refugees from the Middle East, they are not the only significant source of migration to the European continent. For several decades, migration flows within Europe have also been salient. In the last decades, the enlargement of the European Union to the East has contributed to a significant increase in East–West migration, while the recent economic crisis has amplified incentives for the Southern Europeans to migrate to the North of Europe. Similarly, in the past, wealthier European countries resorted to recruitment of foreign labor force from more peripheral European regions and accepted significant numbers of asylum seekers from the countries on the other side of the Iron curtain or refugees fleeing wars in the former Yugoslavia or deadly ethnic conflicts in Turkey. The questions this study addressed were whether and how immigrants' perceptions of the host countries contribute to their life satisfaction. Since host countries are often assessed through the lenses of the sending countries and are presumably indirectly compared to the latter, considering relative levels of satisfaction with host and sending country institutions would be a rather meaningful approach. Hence, in the current study we examined the role of both the absolute and the relative levels of satisfaction with host countries' health and education systems, the functioning of their democracies and the state of their economies on immigrants' overall life satisfaction.

In accordance with the first hypothesis, out of the four domains related to the performance of the host country institutions, satisfaction with economy proved to be the strongest correlate of individual life satisfaction among European immigrants. In support of the second hypothesis, the study maintained that satisfaction with the countries' economies correlates particularly strongly with the overall life satisfaction among immigrants from Turkey, Eastern and Southern Europe. Such a pattern is especially pronounced if we refer to the relative levels of satisfaction with the functioning of economy, which accords with our third hypothesis. In other words, if immigrants at all compare their host and home countries when assessing institutional features of host countries, relative gains in satisfaction due to well-functioning host country institutions are shown to be associated with significantly higher levels of overall life satisfaction among immigrants from Turkey, Eastern and Southern European countries than among immigrants from the rest of Europe. This implies that—in relative terms—migration from countries with presumably worse to countries with better functioning economies and—associated with that—higher levels of perceived satisfaction with host country economies contribute considerably to immigrants' overall satisfaction with life.

In addition to the economy, other features of host societies also matter, but to a considerably lesser extent. Our study showed that perceptions of the state of democracy matter in both absolute and relative terms for immigrants from Turkey, Eastern Europe and Ireland. The state of education system matters for the overall life satisfaction of immigrants from Ireland/UK and Eastern European countries, whereas the state of health services plays a significant role in overall life satisfaction of immigrants from continental and Eastern Europe. Taken together, we can conclude that Eastern Europeans are an immigrants group, for which well-functioning institutions and economy are particularly important for overall life satisfaction.

At large, our results echo major conclusions from Böhnke's (2008) study; namely, perceptions of societies differ in their strength as a determinant of life satisfaction across European countries depending on the level of these countries' development. Our study takes this idea further and shows that individuals from economically prosperous regions with a functioning democracy and efficiently operating systems of public goods tend to put less emphasis on the host country's institutional features when assessing their life satisfaction. If immigrants stem from countries that perform less well—economically or otherwise, the host country's economic and other conditions play a more substantial part in their life satisfaction, even if they are relocated to better-off host societies. Immigrants from Turkey, Eastern and Southern Europe are aware that their life chances depend quite strongly on the improvements—most importantly, in the economic dimension—associated with their migration move.

Another interpretation is possible and might be related to the economic recession European societies endured between 2008 and 2014. Indeed, in this period the bulk of the European countries were strongly affected by first the economic and then the Euro crisis. Concerns about the functioning of countries' financial and labor markets could buttress the finding that immigrants of working age prioritize economic well-being when defining their overall life satisfaction. If it were so, then a similar trend should be observed among socio-demographically comparable native-born populations in the respective host countries. Additional analyses indeed show that satisfaction with the economy is also the most important correlate of individual overall life satisfaction among natives, but the magnitude of beta-coefficients for the majority is considerably lower than it is among the immigrant populations (results are not presented here but available upon request). Further, re-analyses of the data once dropping ESS waves 4 and 5 suggest no substantial change in the pattern of association between perceptions of the state of economy and general life satisfaction among immigrants. If anything, effects of economy become somewhat stronger for immigrants from the UK/Ireland and Turkey, whereas they become somewhat weaker for immigrants from Nordic countries. This suggests that our finding of a paramount significance of economy for immigrants' life satisfaction is not solely driven by the financial and Euro crises catching eye of Europeans in the period under observation.

Our finding that immigrants in many cases assess host-country institutions more positively than both stayers and—even more pronouncedly—than the native-born of the destination countries can be related to the selectivity of migrants. Research has consistently shown that immigrants might be selective on a number of unobserved characteristics, such as immigrant optimism or more positive attitudes to life. If immigrants are generally happier than stayers, which some research tends to suggest—albeit not always consistently (Bartram, 2013, 2015; Akay et al., 2017; Hendriks and Bartram, 2018)—, this might explain their more positive assessment of countries' structural and institutional characteristics, but cannot explain the variation across immigrant groups and countries of destination. Additional assumptions might be needed to elucidate why divergent patterns of evaluations are present. For example, why are immigrants from continental countries in Eastern Europe less satisfied with economy when potentially comparing themselves to their home countries, but are more satisfied than the native-born in the Eastern European countries? Or, on the contrary, why are UK/Irish immigrants in Nordic countries particularly satisfied with economy when comparing themselves to the situation back home, but are considerably less satisfied when comparing it to the native-born of the Northern European countries? Without disregarding the role immigrant selectivity might play in migration decisions and the subsequent outcomes related to life satisfaction, we contend that assessments of the objective state of host-country institutions cannot be primarily driven by immigrants' self-selection patterns.

Unfortunately, the cross-sectional nature of the ESS data does not allow an in-depth investigation about the mechanisms behind the observed associations. The reverse causality might be of concern: individuals who are more satisfied with their lives might be more likely to report satisfaction with the host country institutions. Yet, this cannot explain the extent of variation regarding the importance of various host country features: immigrants of different origins attach different meanings to a well-functioning economy and democracy in their host countries as well as to the state of their education and health systems. Heterogeneous effects across immigrant populations, related to differences in needs and/or aspirations, might also be a venue for future research. For example, older or chronically ill individuals might attach significant importance to health services, while parents of smaller children value a well-functioning educational system more. These issues open multiple opportunities for further investigations.

## Author Contributions

All authors listed have made a substantial, direct and intellectual contribution to the work, and approved it for publication.

### Conflict of Interest Statement

The authors declare that the research was conducted in the absence of any commercial or financial relationships that could be construed as a potential conflict of interest.

## Footnotes

^1^The importance of multiple comparison frames among immigrants has been emphasized in earlier studies (Falk and Knell, 2004; Gelatt, 2013).

^2^The European Social Survey is a unique cross-sectional survey that covers a large number of European countries and allows for comparisons both between migrants and non-migrants. It is repeated every second year since 2002, and said to be representative of the population aged 15 or older.

^3^We excluded Turkey as a destination country, focusing solely on Turkish migrants residing within rest of Europe.

^4^Figures 1ab-4ab present average satisfaction levels with the functioning of institutions in respective country groups for immigrant groups with at least 50 observations. For differences between Ireland and the UK, consult Table A.1 in the Appendix.

^5^This does not refer to the relative levels of satisfaction with the state of health system among Eastern Europeans.

^6^A single exception is the difference between the respective coefficient for Irish/British and immigrants from the continental Europe, but the coefficients are different solely at the 10% level.

## References

[B1] Akay A. Bargain O. Zimmermann K. F. (2017). Home sweet home? Macroeconomic conditions in home countries and the well-being of migrants. J. Hum. Resour. 52, 351–373. 10.3368/jhr.52.2.0115-6900R1

[B2] Allardt E. (1976). Dimensions of welfare in a comparative scandinavian study. Acta Sociol. 19, 227–239. 10.1177/000169937601900302

[B3] Andrews F. M. Withey S. B. (1976). Social Indicators of Well- Being: Americans' Perceptions Of Life Quality. New York, NY: Plenum Press.

[B4] Argyle M. (1999). Causes and correlates of happiness, in Well-Being: The Foundations of Hedonic Psychology, eds Kahneman D. Diener E. Schwarz N. (New York, NY: Russell Sage Foundation), 353–373.

[B5] Bade K. J. (2004). Legal and illegal immigration into Europe: experiences and challenges. Eur. Rev. 12, 339–375. 10.1017/S1062798704000316

[B6] Bartram D. (2011). Economic migration and happiness: comparing immigrants' and natives' happiness gains from income. Soc. Indic. Res. 103, 57–76. 10.1007/s11205-010-9696-2

[B7] Bartram D. (2013). Happiness and 'Economic migration': a comparison of eastern european migrants and stayers. Migrat. Stud. 1, 156–175. 10.1093/migration/mnt006

[B8] Bartram D. (2015). Inverting the logic of economic migration: happiness among migrants moving from wealthier to poorer countries in Europe. J. Happiness Stud. 16, 1211–1230. 10.1007/s10902-014-9554-z

[B9] Baykara-Krumme H. Platt L. (2018). Life satisfaction of migrants, stayers and returnees: reaping the fruits of migration in old age? Ageing Soc. 38, 721–745. 10.1017/S0144686X16001227

[B10] Böhnke P. (2008). Does society matter? Life satisfaction in the enlarged Europe. Soc. Indicat. Res. 87, 189–210. 10.1007/s11205-007-9169-4

[B11] Campbell A. Converse P. E. Rodgers W. L. (1976). The Quality of American Life: Perceptions, Evaluations, and Satisfactions. New York, NY: Russel Sage Foundation.

[B12] Castles S. De Haas H. Miller M. J. (2014). The Age of Migration: International Population Movements in the Modern World. Basingstoke: Palgrave Macmillan.

[B13] Clark A. E. (2003). Unemployment as a social norm: psychological evidence from panel data. J. Labor Econ. 21, 323–351. 10.1086/345560

[B14] Diener E. Suh E. M. (1999). National differences in subjective well-being, in Well-Being: The Foundations of Hedonic Psychology, eds Kahneman D. Diener E. Schwarz N. (New York, NY: Russel Sage Foundation), 434–450.

[B15] Dorn D. Fischer J. A. V. Kirchgässner G. Sousa- Poza A. (2007). Is it culture or democracy? The impact of democracy and culture on happiness. Soc. Indic. Res. 82, 505–526. 10.1007/s11205-006-9048-4

[B16] Dorn D. Fischer J. A. V. Kirchgässner G. Sousa-Poza A. (2008). Direct democracy and life satisfaction revisited: new evidence for Switzerland. J. Happiness Stud. 9, 227–255. 10.1007/s10902-007-9050-9

[B17] Easterlin R. A. (1973). Does money buy happiness? Pub. Int. 30, 3–10.

[B18] Fahey T. Smyth E. (2004). The link between subjective well-being and objective conditions in european societies, in European Values at the Turn of the Millennium, eds Arts W. Halman L. (Leiden: Brill), 57–80.

[B19] Falk A. Knell M. (2004). Choosing the joneses: endogenous goals and reference standards. Scand. J. Econ. 106. 417–435. 10.1111/j.0347-0520.2004.00370.x

[B20] Fassmann H. Münz R. (1992). Patterns and trends of international migration in Western Europe. Popul. Dev. Rev. 18, 457–480. 10.2307/1973654

[B21] Fassmann H. Münz R. (1994). European East-West Migration, 1945-1992. Int. Migr. Rev. 28, 520–538. 10.1177/019791839402800305 12345793

[B22] Festinger L. (1954). A theory of social comparison processes. Hum. Relat. 7, 117–140. 10.1177/001872675400700202

[B23] Fleischmann F. Dronkers J. (2010). Unemployment among immigrants in european labour markets: an analysis of origin and destination effects. Work Employ. Soc. 24, 337–354. 10.1177/0950017010362153

[B24] Frey B. S. Stutzer A. (2003). Direct democracy: designing a living constitution. Zurich IEER Working Paper No. 167.

[B25] Gelatt J. (2013). Looking down or looking up: status and subjective well-being among asian and latino immigrants in the United States. Int. Migr. Rev. 47. 39–75. 10.1111/imre.12013 24151347 PMC3800169

[B26] Gorodzeisky A. Semyonov M. (2017). Labor force participation, unemployment and occupational attainment among immigrants in West European Countries. PLoS ONE 12:e0176856. 10.1371/journal.pone.0176856 28475632 PMC5419508

[B27] Haller M. Hadler M. (2006). How social relations and structures can produce happiness and unhappiness: an international comparative analysis. Soc. Indic. Res. 75, 169–216. 10.1007/s11205-004-6297-y

[B28] Hansen R. (2002). Globalization, embedded realism, and path dependence: the other immigrants to Europe. Comp. Polit. Stud. 35, 259–283. 10.1177/0010414002035003001

[B29] Harris J. R. Todaro M. P. (1970). Migration, unemployment and development: a two sector analysis. Am. Econ. Rev. 60, 126–142.

[B30] Hatton T. J. (2004). Seeking asylum in Europe. Econ. Policy 19, 6–62. 10.1111/j.1468-0327.2004.00118.x

[B31] Heath A. F. Cheung S. Y. (2007). Unequal Chances: Ethnic Minorities in Western Labour Markets. Oxford: Oxford University Press.

[B32] Heath A. F. Rothon C. Kilpi E. (2008). The second generation in western europe: education, unemployment and occupational attainment. Annu. Rev. Sociol. 34, 211–235. 10.1146/annurev.soc.34.040507.134728

[B33] Hendriks M. (2015). The happiness of international migrants: a review of research findings. Migr. Stud. 3, 343–369. 10.1093/migration/mnu053

[B34] Hendriks M. Bartram D. (2016). Macro-conditions and Immigrants' happiness: is moving to a wealthy country all that matters? Soc. Sci. Res. 56, 90–107. 10.1016/j.ssresearch.2015.11.006 26857174

[B35] Hendriks M. Bartram D. (2018). Bringing happiness into the study of migration and its consequences: what, why, and how? J. Immigr. Refugee Stud. 10.1080/15562948.2018.1458169

[B36] Hsieh C. M. (2017). Health, quality of homecare service and quality of life: a case of frail older immigrant adult. Soc. Indicat. Res. 134, 711–723. 10.1007/s11205-016-1442-y

[B37] Inglehart R. Klingemann H. D. (2000). Genes, culture, and happiness, in Subjective Well-Being across Cultures, eds Diener E. Suh E. M. (Cambridge, MA: MIT Press), 165–183.

[B38] Jones E. E. Davis K. E. (1965). From acts to dispositions: The attribution process in person perception, in Advances in Experimental Social Psychology, Vol. 2, ed L. Berkowitz (New York, NY: Academic Press), 219–266. 10.1016/S0065-2601(08)60107-0

[B39] Kamberi E. Martinovic B. Verkuyten M. (2015). Life satisfaction and happiness among the roma in central and southeastern Europe. Soc. Indic. Res. 124, 199–220. 10.1007/s11205-014-0783-7

[B40] Kelley H. H. (1971). Attribution in social interaction, in Attribution: Perceiving the Causes in Behavior, eds Jones E. E. Kanouse D. E. Kelley H. H. Nisbett R. E. Valins S. Weiner B. (Morristown, NJ: General Learning), 1–26.

[B41] Khoudja Y. Platt L. (2018). Labour market entries and exits of women from different origin countries in the UK. Soc. Sci. Res. 69, 1–18. 10.1016/j.ssresearch.2017.10.003 29169530

[B42] Kogan I. (2006). Labor markets and economic incorporation among recent immigrants in Europe. Soc. Forces 85, 697–721. 10.1353/sof.2007.0014

[B43] Kogan I. (2007). A study of immigrants' employment careers in west germany using the sequence analysis technique. Soc. Sci. Res. 36, 491–511. 10.1016/j.ssresearch.2006.03.004

[B44] Kogan I. Shen J. Siegert M. (2018). What makes a satisfied immigrant? Host-country characteristics and immigrants' life satisfaction in eighteen european countries. J. Happin. Stud. 19, 1783–1809. 10.1007/s10902-017-9896-4

[B45] Lee E. S. (1966). A theory of migration. Demography 3, 47–57. 10.2307/2060063

[B46] Michalos A. C. (1985). Multiple disrepanies theory (MDT). Soc. Indicat. Res. 16, 347–413. 10.1007/BF00333288

[B47] Piore M. J. (1979). Birds of Passage: Migrant Labour in Industrial Societies. Cambridge: Cambridge University Press. 10.1017/CBO9780511572210

[B48] Schyns P. (1998). Crossnational differences in happiness: economic and cultural factors explored. Soc. Indicat. Res. 43, 3–26. 10.1023/A:1006814424293

[B49] Shin D. C. Johnson D. M. (1978). Avowed happiness as an overall assessment of the quality of life. Soc. Indicat. Res. 5, 475–492. 10.1007/BF00352944

[B50] Siegert M. (2013). Die Zufriedenheit der Migranten in Westdeutschland: Eine empirische Analyse. Wiesbaden: Springer.

[B51] Van Mol C. de Valk H. (2016). Migration and immigrants in europe: a historical and demographic perspective, in Integration Processes and Policies in Europe, IMISCOE Research Series, eds Garcés-Mascare-as B. Penninx R. (Cham: Springer), 31–55. 10.1007/978-3-319-21674-4_3

[B52] Van Tubergen F. Maas I. Flap H. (2004). The economic incorporation of immigrants in 18 western societies: origin, destination, and community effects. Adv. Sci. Res. 69, 704–727. 10.1177/000312240406900505

[B53] Veenhoven R. (1997). Advances in understanding happiness. Revue Québécoise de Psychol. 18, 29–74.

[B54] Veenhoven R. (2000). Freedom and happiness: a comparative study in forty-four nations in the early 1990s, in Culture and Subjective Well-Being, eds Diener E. Suh E. M. (Cambridge, MA: MIT Press), 257–288.

[B55] Zipf G. K. (1946). The P1P2/D hypothesis: on the intercity movement of persons. Am. Soc. Rev. 11. 677–686. 10.2307/2087063

