# Supplementary material for: It's the Economy! Perceptions of Host-Countries' Institutions and Individual Life Satisfaction of Intra-European Migrants
Source: Front Sociol. 2019 May 15;4:42. doi: 10.3389/fsoc.2019.00042 (PMC8022558; doi:10.3389/fsoc.2019.00042)
Supplement: Supplementary file 1 [file Table_1.docx]

Appendix

Figure A.1: Satisfaction with the state of economy, relative to the native-born in the host country

Figure A.2: Satisfaction with the state of democracy, relative to the native-born in the host country

Figure A.3: Satisfaction with the state of education system, relative to the native-born in the host country

Figure 4b: Satisfaction with the state of health system, relative to the native-born in the host country

Table A.1:

|  | Ireland | | UK | |
| --- | --- | --- | --- | --- |
|  | Mean | SD | Mean | SD |
| Economy absolute | 4.15 | 2.46 | 3.72 | 2.40 |
| Economy relative to stayers | 0.71 | 2.58 | -0.13 | 2.29 |
| Democracy absolute | 4.86 | 2.62 | 5.22 | 2.41 |
| Democracy relative to stayers | -0.24 | 2.69 | 0.17 | 2.37 |
| Education system absolute | 5.92 | 1.85 | 6.18 | 2.06 |
| Education system relative to stayers | -0.26 | 1.89 | 0.49 | 2.05 |
| Health system absolute | 6.01 | 2.48 | 4.79 | 2.55 |
| Health system relative to stayers | 1.98 | 2.50 | -1.08 | 2.55 |

Source: ESS 2008-2016 (rounds 4-8), weighted data, authors’ calculations

Table A.2: Satisfaction with host-country institutions in comparison with the native-born, selected coefficients from OLS regressions predicting life satisfaction among immigrants

|  | Relative to natives in the country of current residence | | | |
| --- | --- | --- | --- | --- |
|  | b | se | beta | Sign. dif. |
| *(a) Continental* |  |  |  |  |
| State of economy | 0.23*** | (0.03) | 0.22 | b |
| State of democracy | 0.03 | (0.03) | 0.03 | c, *f* |
| State of education | 0.01 | (0.03) | 0.01 |  |
| State of health system | 0.05^+^ | (0.03) | 0.05 |  |
| *(b) Northern* |  |  |  |  |
| State of economy | 0.10^+^ | (0.05) | 0.09 | a, d, e, f |
| State of democracy | 0.09^+^ | (0.05) | 0.06 |  |
| State of education | 0.03 | (0.05) | 0.03 |  |
| State of health system | 0.08 | (0.05) | 0.08 |  |
| *(c) Ireland/UK* |  |  |  |  |
| State of economy | 0.12** | (0.04) | 0.11 | a, d, e, f |
| State of democracy | 0.14*** | (0.04) | 0.14 | a*, d,* e |
| State of education | 0.07^+^ | (0.04) | 0.07 |  |
| State of health system | 0.01 | (0.03) | 0.01 | *d* |
| *(d) Eastern* |  |  |  |  |
| State of economy | 0.27*** | (0.02) | 0.26 | b, c |
| State of democracy | 0.07 | (0.02) | 0.07 |  |
| State of education | 0.01 | (0.03) | 0.01 |  |
| State of health system | 0.05 | (0.03) | 0.05 |  |
| *(e) Southern* |  |  |  |  |
| State of economy | 0.28*** | (0.04) | 0.27 | b, c |
| State of democracy | 0.04 | (0.04) | 0.04 | *c* |
| State of education | 0.08^+^ | (0.04) | 0.08 |  |
| State of health system | 0.01 | (0.04) | 0.01 | *d*, |
| *(f) Turkey* |  |  |  |  |
| State of economy | 0.31*** | (0.04) | 0.30 | b, c |
| State of democracy | 0.14** | (0.05) | 0.14 | *a* |
| State of education | 0.05 | (0.05) | 0.05 |  |
| State of health system | 0.08^+^ | (0.04) | 0.08 |  |
| N | 5,100 | | | |
| R^2^ | 0.38 | | | |

Source: ESS 2008-2016 (rounds 4-8), weighted data, authors’ calculations

Notes: (1) ^+^ p<0.10, * p < 0.05, ** p < 0.01, *** p < 0.001; (2) Letters in column 4 and 8 indicate whether differences of the group shown are significant compared to the groups indicated by a letter; letters in bold pertain to coefficients significant solely at 10%-level. (3) Control variables included in the model are: age, age squared, gender, family status, presence of children, employment status, YSM, citizenship status, income, language spoken, social contacts, safety situation, subjective health, religiosity, minority status, origin groups main effects, country of residence fixed effects, year of interview fixed effects.
